# Constraint‐based metabolic modeling reveals metabolic properties underpinning the unprecedented growth of *Chlorella ohadii*

**DOI:** 10.1111/nph.70528

**Published:** 2025-09-05

**Authors:** Fayaz Soleymani, Sandra Marcela Correa, Marius Arend, Niayesh Forghanisardaghi, Haim Treves, Zahra Razaghi‐Moghadam, Zoran Nikoloski

**Affiliations:** ^1^ Systems Biology and Mathematical Modeling Group Max Planck Institute of Molecular Plant Physiology 14476 Potsdam Germany; ^2^ Bioinformatics Department, Institute of Biochemistry and Biology University of Potsdam 14476 Potsdam Germany; ^3^ Department of Biology RPTU 67663 Kaiserslautern Germany

**Keywords:** *Chlorella ohadii*, *de novo* model reconstruction, gene targets, genome‐scale metabolic model, growth improvement, metabolic model comparison

## Abstract

- Comparative molecular and physiological analyses of organisms from one taxonomic group grown under similar conditions offer a strategy to identify gene targets for trait improvement. While this strategy can also be performed *in silico* using genome‐scale metabolic models for the compared organisms, we continue to lack solutions for the *de novo* generation of such models, particularly for eukaryotes.
- To facilitate model‐driven identification of gene targets for growth improvement in green algae, here we present a semiautomated platform for *de novo* generation of genome‐scale algal metabolic models. We deployed this platform to reconstruct an enzyme‐constrained, genome‐scale metabolic model of *Chlorella ohadii*, the fastest growing green alga reported to date, and validated the growth predictions in experiments under three growth conditions. We also proposed a computational strategy to identify targets for growth improvement based on flux analyses.
- Extensive flux‐based comparative analyses using all existing models of green algae resulted in the identification of potential targets for growth improvement not only in standard but also in extreme light conditions, where *C. ohadii* still exhibits exceptional growth.
- Our findings indicate that the developed platform provides the basis for the generation of pan‐genome‐scale metabolic models of algae.

## Introduction

The expected increase in the global population requires urgent innovation to address the limitations and shortcomings of current industrial and agricultural practices that directly rely on photosynthetic organisms. Synthetic biology approaches that aim to redesign metabolic networks not only bear great potential for optimization of future crops (South *et al*., 2019), but also pose feasibility challenges (Bar‐Even, 2018). Algae, particularly species from extreme habitats, represent an important genetic resource for raising gene targets for biotechnological and agricultural applications. This is due to their remarkable metabolic flexibility that has contributed to sustaining growth even under very harsh environments (Georgianna & Mayfield, 2012). A comparative study of slow and fast‐growing algal species grown under similar conditions can be used to identify potential gene targets that underpin the difference in growth.

The green alga *Chlorella ohadii*, recently isolated from one of the harshest habitats, the Israeli Negev desert (Treves *et al*., 2013), is an attractive candidate for such a comparative approach as it exhibits the fastest doubling time (*c*. 1.4 h) ever reported for any eukaryotic photosynthetic organism and remarkable photosynthetic rates per Chl (600–700 μmol O_2_ mg^−1^ Chl h^−1^) as well as extreme photodamage resistance (Treves *et al*., 2016, 2017). Further, under optimal conditions, this alga exhibits an unprecedented doubling time and per‐Chl photosynthetic rates that are four‐ and threefold faster, respectively, than those of the model green alga *Chlamydomonas reinhardtii* (Mettler *et al*., 2014; Ananyev *et al*., 2017; Treves *et al*., 2017). It has been shown that coordinated metabolic shifts and diverse pathways contribute to the unprecedented growth of *C. ohadii* (Treves *et al*., 2017). However, systematic experimental analyses of all processes and underlying genes that contribute to the differences in growth are an enormous undertaking. While the existing omics technologies can be used to address this question, the relation of the data read‐outs to growth remains challenging to assess without samples from multiple experiments; these data enable the development of machine learning models for growth in terms of the generated omics data.

Genome‐scale metabolic modeling provides an alternative to molecular profiling for the identification of gene targets for trait improvement, as it allows both integration of diverse data sets and prediction of specific growth rates and reaction fluxes. Genome‐scale metabolic models have already been reconstructed for several microalgae (Tibocha‐Bonilla *et al*., 2018), including *C. reinhardtii* (Chang *et al*., 2011; Imam *et al*., 2015), *Chlorella vulgaris* (Zuñiga *et al*., 2016, 2018), and *Chlorella variabilis* (Juneja *et al*., 2016). These models have been used with modeling approaches that incorporate constraints, like metabolic steady state and measured exchange fluxes, to predict flux phenotypes by assuming optimization of a cellular task (e.g. growth in flux balance analysis (FBA)) (Nikoloski *et al*., 2015). Furthermore, the consideration of enzyme turnover numbers (i.e. catalytic rates) and total protein content in protein‐constrained models has facilitated more accurate predictions of growth, fluxes, and allocation of enzyme abundance (Arend *et al*., 2023; Ferreira *et al*., 2024).

However, all genome‐scale metabolic models of green microalgae, with one exception, have been reconstructed using the metabolic model of *C. reinhardtii* as a template. While the template‐based approach to model reconstruction has provided important insights, it can bias the extracted metabolic network since pathway and reaction inclusion are solely based on those present in the template. For instance, the comparative approach of the resulting flux predictions can result in false targets explaining the growth differences between the compared algae. In contrast to the template‐based approach, the metabolic model *C. variabilis* (Juneja *et al*., 2016) relies on the ModelSEED database (Seaver *et al*., 2021). However, usage of a single database of biochemical reactions has been shown to result in a model that includes only a portion of all annotated genes, even in the case of prokaryotes (Wendering & Nikoloski, 2022; Hsieh *et al*., 2024). Usage of multiple databases of biochemical reactions can readily address this problem.

To facilitate a model‐driven approach to raising targets for growth improvement as well as to allow semiautomated generation of algal models, here we present an innovative platform for *de novo* generation of genome‐scale algal metabolic models. We note that the procedures for compartmentalization and for removal of thermodynamically infeasible cycles (TICs) are fully automated; however, the former relies on predictions from machine learning models and necessitates human intervention to avoid propagation of errors from protein localization predictions. We deploy this platform to reconstruct a genome‐scale metabolic model of *C. ohadii*. Extensive comparison of structural and functional differences between existing metabolic models of algal species showcases the capacity of this platform to generate models resulting in accurate predictions that we use to raise targets for growth improvement.

## Materials and Methods

The genome‐scale metabolic model (GEM) reconstruction pipeline includes steps mainly following a well‐established protocol (Thiele & Palsson, 2010). These steps are categorized into five principal stages, namely draft reconstruction, biomass reaction determination, gap‐filling, compartmentalization, and an additional step of creating enzyme‐constrained GEM. Here, we describe the relevant points in each phase, with principal novelty regarding compartmentalization.

### Draft reconstruction

The draft GEM reconstruction was conducted based on the annotated genome of *Chlorella ohadii* using the RAVEN toolbox (Wang *et al*., 2018).

The draft reconstruction pipeline is based on the annotated genome of the green alga *Chlorella ohadii* (NCBI Taxonomy ID 2649997, reference genome ASM2502687v1; Treves *et al*., 2013), as input. The corresponding GenBank assembly data GCA_025026875.1 contains 10 866 protein‐coding genes. We used the RAVEN toolbox (Wang *et al*., 2018) v.2.8.4 with the purpose of *de novo* reconstruction of the draft GEM.

The input FASTA file is utilized to reconstruct draft networks based on the two databases, that is, KEGG (Kanehisa, 2002) and MetaCyc (Caspi *et al*., 2020). KEGG‐based draft reconstruction queries protein sequences on pre‐trained Hidden Markov Models (HMMs) to find similarities with annotated genes in the database. On the other hand, MetaCyc‐based draft reconstruction employs Blastp (Altschul *et al*., 1990) for querying protein sequences on curated enzymes in the database. Consequently, two separate models based on KEGG and MetaCyc are combined in order to have one unified GEM draft.

### Biomass reactions for different growth conditions

In this step, the chemical composition of the cell is determined by taking advantage of both experimental data and genome‐based estimations to build four biomass reactions for different growth conditions.

To simulate growth, we consider three different growth conditions, namely photoautotrophic (CO_2_ as a carbon source in the presence of light), mixotrophic (CO_2_ and acetate as carbon sources in the presence of light), and heterotrophic (acetate as the single carbon source in the absence of light). The photoautotrophic condition was simulated for two different light intensities, 100 μmol photons m^−2^s^−1^ and 3000 μmol photons m^−2^ s^−1^, which results in two respective biomass reactions. In the following, for simplification, we refer to the four biomass reactions as biomass_auto_100, biomass_auto_3k, biomass_mixo, and biomass_hetero.

The biomass content was categorized into six main classes: DNA, RNA, proteins, carbohydrates, Chl, and lipids/fatty acids. For biomass_auto_100 and biomass_auto_3k, the absolute weight of proteins, DNA, Chl*a*, Chl*b*, starch, galactose, glucose, mannose, and sucrose, based on the weight per dry weight of the cell, was obtained from published experimental data (Treves *et al*., 2022). However, the fraction of lipids/fatty acids was mapped from the proportions of corresponding metabolites in the well‐curated GEM of *C. reinhardtii* iCre1355 (Imam *et al*., 2015). In addition, in the absence of data from *C. ohadii*, for the mixotrophic and heterotrophic conditions, the biomass composition from the iCre1355 model was used. Finally, the ratios for all the class proportions were used to ensure that they sum to 1 g g^−1^ DW of the cell. The details of the cell composition under each growth condition are provided in Supporting Information Table S1.

After estimating the composition of the biomass reaction for each growth condition, the coefficients for each biomass precursor were determined. The molar percentages of each amino acid, deoxynucleotide triphosphates, and nucleotide triphosphates were calculated based on the genome of *C. ohadii*. To estimate the coefficients of carbohydrates, Chl, and lipids/fatty acids, we used published experimental data; where data were not available, we used rescaled coefficients from iCre1355. The details of the stoichiometric coefficients of the biomass precursors under each growth condition are available in Table S2.

The media composition was considered in the model by adding exchange reactions for H_2_O, H^+^, Pi, NH_4_, SO_4_, Fe^+2^, Mg^+2^, and O_2_, and for condition‐specific exchange reactions, namely light, acetate, and CO_2_.

### Gap‐filling

Gap‐filling was performed with the draft reconstruction with the goal to produce biomass precursors and consequently predict specific growth rates using FBA (Orth *et al*., 2010). Details about gap‐filling are provided in Methods S1.

### Compartmentalization

Compartmentalization is performed by assigning a compartment for each reaction regarding the predictions based on the associated enzymes, followed by mixed integer linear programming (MILP) to minimize the number of added reactions to the model. This procedure ensures model functionality by replicating reactions across relevant compartments based on GPR rules and predicted enzyme subcellular localization, while also introducing the minimal necessary set of transport reactions. Details about the steps of the compartmentalization procedure are provided in Methods S2.

### Elimination of thermodynamically infeasible cycles

Thermodynamically infeasible cycles (TICs), also known as Type III loops, are defined as cycles that can carry flux in the absence of any exchange reactions. We propose a novel approach to remove the TICs in the GEM, based on the MILP, which removes a minimal set of reactions, while maintaining biomass. In summary, we identified the set of all reactions involved in TICs by solving a linear programming (LP) problem. We then eliminated the minimal subset of these reactions by solving an MILP problem in such a way that they cannot carry flux in the absence of uptake reactions, while simultaneously ensuring biomass production (see Methods S3).

### Simulation of growth conditions

The lower bounds for the uptake reactions for eight metabolites, namely: H_2_O (10 mmol g^−1^ DW h^−1^), H^+^ (10 mmol g^−1^ DW h^−1^), Pi (10 mmol g^−1^ DW h^−1^), NH_4_ (10 mmol g^−1^ DW h^−1^), SO_4_ (10 mmol g^−1^ DW h^−1^), Fe^+2^ (10 mmol g^−1^ DW h^−1^), Mg^+2^ (10 mmol g^−1^ DW h^−1^), and O_2_ (10 mmol g^−1^ DW h^−1^), were set across the three simulated growth conditions. In addition, simulations for the photoautotrophic low light and mixotrophic conditions were performed at the light intensity set at 100 μmol photons m^−2^s^−1^, while the photoautotrophic high‐light condition was set at 3000 μmol photons m^−2^s^−1^. Further, heterotrophic and mixotrophic conditions allowed the uptake of acetate equal to 2 mmol g^−1^ DW h^−1^, in line with estimations from experiments (Treves *et al*., 2022). We note that since the predicted specific growth rates varied significantly across algal species, we displayed them on a logarithmic scale.

### Constructing enzyme‐constrained model and atom mappings

To construct the enzyme‐constrained model, GECKO (Sánchez *et al*., 2017) was used based on the predicted $k_{\mathrm{cat}}$ values from TurNuPred (Kroll *et al*., 2023). First, for each enzymatic reaction and for each enzyme, the protein sequence, substrates, and products were extracted. Consequently, these data were used as the input for a deep learning $k_{\mathrm{cat}}$ prediction tool named TurNuPred. Then, the GECKO formulation was applied to the model using the predicted $k_{\mathrm{cat}}$ values to obtain the enzyme‐constrained genome‐scale metabolic network. The total enzyme content was obtained from published experimental measurements of *C. ohadii* (Treves *et al*., 2022). Finally, atom mapping of 1926 out of 3230 (> 50%) reactions of iCO1515 GEM was obtained based on the deep learning method named LocalMapper (Chen *et al*., 2024).

## Results

### Pipeline for generation of a compartmentalized GEM of *C. ohadii*

**Fig. 1:**
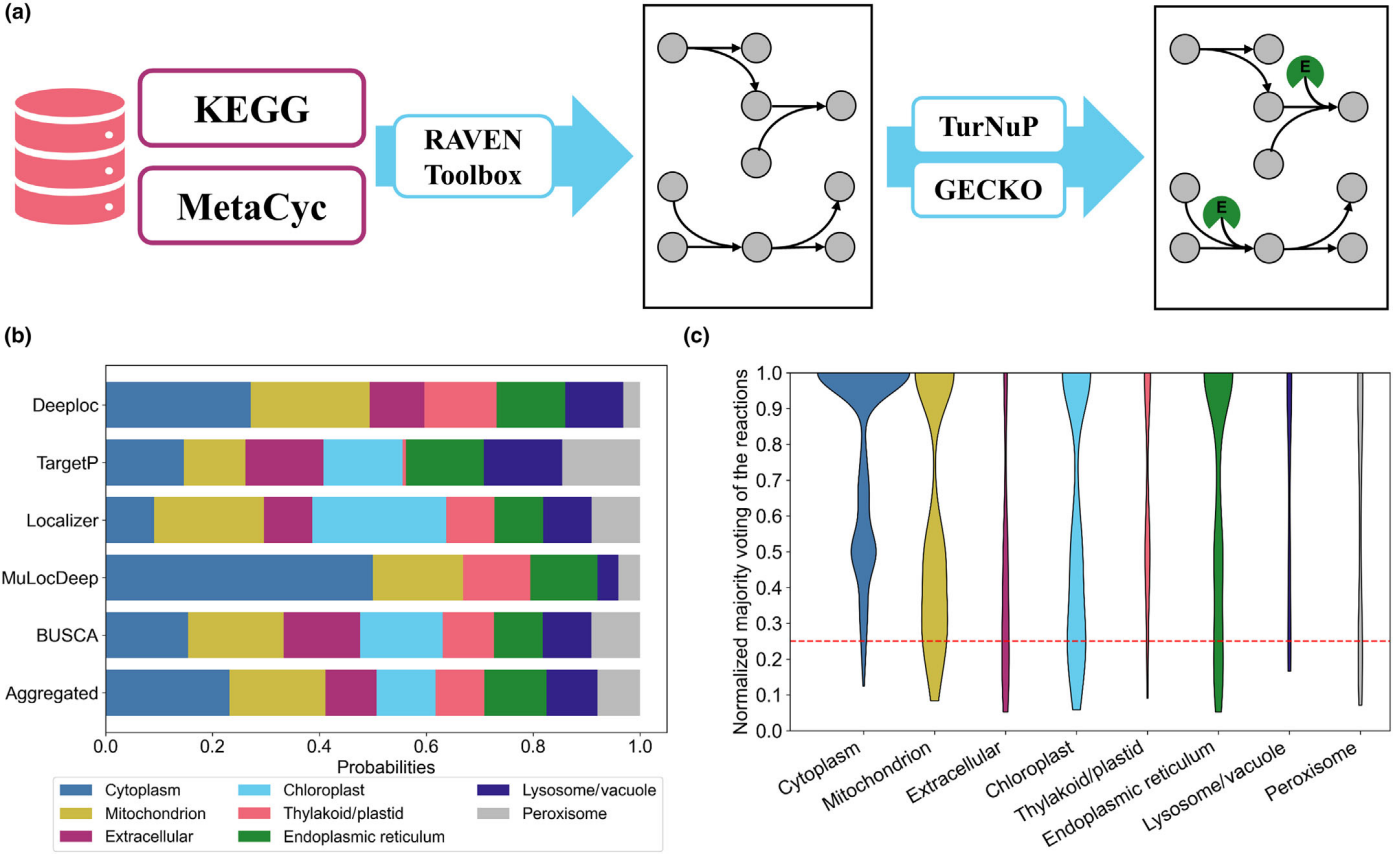
Highlights of the model reconstruction procedure and the distribution of proteins and reactions per compartments. (a) Draft reconstruction based on the annotated genome of *Chlorella ohadii*, using Kegg (Kanehisa, 2002) and MetaCyc (Caspi *et al*., 2020) databases with the RAVEN toolbox (Wang *et al*., 2018). The figure illustrates the procedure for constructing the enzyme‐constraint genome‐scale metabolic model (GEM) from the conventional GEM employing $k_{\mathrm{cat}}$ values predicted from TurNuP (Kroll *et al*., 2023) and integration of enzyme constraints based on GECKO (Sánchez *et al*., 2017). (b) The bar plot displays the distribution of predicted localization of all proteins of the iCO1515 GEM in the annotated genome of *C. ohadii* (Treves *et al*., 2013) in at most eight compartments, represented with different colors (see key), using five prediction tools. (c) The violin plot shows majority voting for all reactions of iCO1515, normalized to the number of genes in their respective gene–protein‐reaction rules. The dashed line presents the threshold of 25% above which a reaction is retained in a given compartment.

The enzyme‐constrained GEM of *C. ohadii*, termed eciCO1515, is based on a draft reconstruction that is successively refined by: (1) gap‐filling, (2) applying newly devised procedures for automated compartmentalization and elimination of TICs, and (3) adding enzymatic constraints (Fig. 1a). We stress that the second and third refinements are, at present, partially or not automated.

The draft reconstruction was obtained based on the annotated genome of *C. ohadii* (Treves *et al*., 2013), employing the RAVEN toolbox (Wang *et al*., 2018) that retrieves reactions associated with homologous genes from two databases, that is, KEGG (Kanehisa, 2002) and MetaCyc (Caspi *et al*., 2020) (see ‘Draft reconstruction’ in the Materials and Methods section). The usage of more than one database of biochemical reactions aims to increase the number of included genes, as stated in the section above. We then performed gap‐filling with the entire KEGG model, followed by a series of manual curation steps, including the semiautomated insertion of a well‐curated adaptation of the Plant Lipid Module to green algae (Córdoba *et al*., 2023) and the creation of a biomass reaction based on measured biomass composition. This allowed us to generate a functional, *uncompartmentalized* model able to simulate growth under three growth conditions, photoautotrophic, mixotrophic, and heterotrophic.

To compartmentalize the model, we used five tools to predict subcellular localization of proteins: Deeploc v.2.0 (Thumuluri *et al*., 2022), TargetP v.2.0 (Armenteros *et al*., 2019), Localizer (Sperschneider *et al*., 2017), MuLocDeep (Jiang *et al*., 2021), and Busca (Savojardo *et al*., 2018) (see Table S3 as well as the Materials and methods section, detailing the criteria used for tool selection). The predictions of these tools placed proteins into at maximum of eight compartments included in the model: cytoplasm, chloroplast, lysosome/vacuole, mitochondrion, thylakoid/plastid, peroxisome, endoplasmic reticulum, and extracellular space. Application of each tool resulted in a tool‐specific probability that a protein is localized in a particular compartment (Fig. 1b). For instance, MuLocDeep placed a protein with the largest probability in the cytoplasm, in contrast to Localizer, which placed it in the chloroplast. Some of these tools, like Deeploc and MuLocDeep, did not predict any proteins to be localized in the chloroplast. To consolidate these predictions, we determined the aggregated probabilities across the five prediction tools (Fig. 1c), relying on the wisdom of the crowd principle (Bühlmann, 2012).

Reactions were placed in specific compartments based on majority voting of the localization predictions using the gene–protein‐reaction (GPR) rules. As a result of this fully automated step, we obtained a distribution of reactions across compartments (Fig. 1c). These distributions were used to generate and validate a compartmentalized model, as detailed in the following section.

### Novel procedure for GEM compartmentalization

**Fig. 2:**
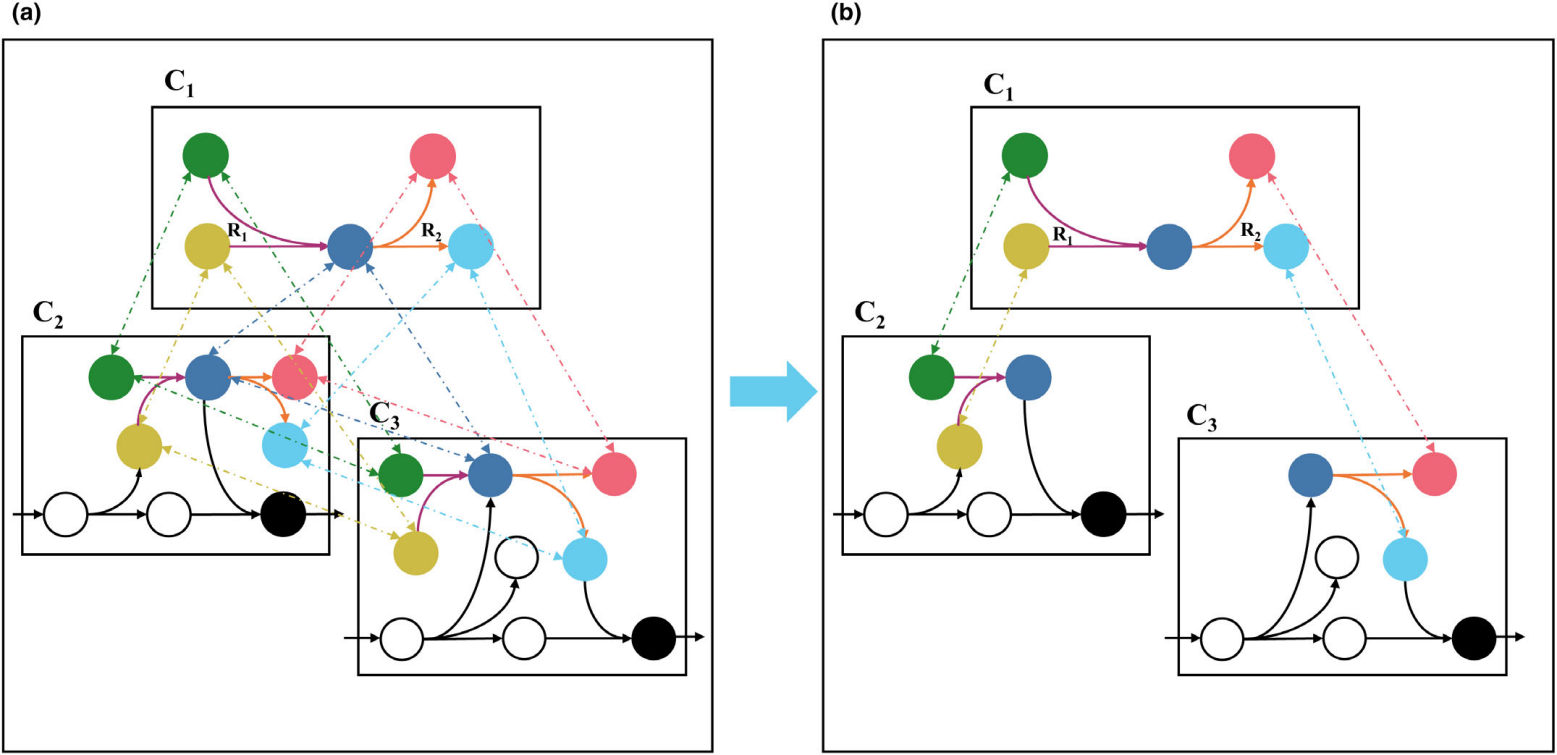
Toy example illustrating the novel compartmentalization method. (a) The toy example illustrates the optimization problem for resolving the compartmentalization. The reactions R_1_ (purple) and R_2_ (orange) are allocated in three compartments, denoted by C_1_, C_2_, and C_3_; all possible transport reactions between the five common (i.e. shared) metabolites, marked in green, pink, red, blue, and cyan, are added to the model. (b) Model after compartmentalization, obtained by minimizing the number of added reactions while ensuring functionality, defined by the ability to produce specific precursors in the compartments, here colored in black. As a result, one instance of the replicated reaction R_2_ is removed from C_2_, and one instance of the replicated reaction R_1_ is removed from C_3_; in addition, the redundant transport reactions are pruned to ensure functionality.

Based on the majority voting procedure for a reaction, we accumulated and normalized the votes so that the reaction appears in all compartments with a number of votes greater than a specified threshold value. As a result, a reaction may be assigned to more than one compartment; we refer to these as *replicated reactions*. Our fully automated procedure for GEM compartmentalization includes three steps: (1) allocating replicated reactions to compartments based on the majority voting results, (2) adding supplementary transport reactions between compartment‐specific pools of a given metabolite, and (3) solving an optimization problem, cast as a mixed‐integer linear program, that retains the smallest set of the replicated and supplementary reactions while ensuring model functionality, given by the ability to simulate the production of biomass precursors (see ‘Compartmentalization’ in the Materials and Methods section, Fig. 2). Therefore, the resulting solutions respect the protein localization while invoking the parsimony principle.

To evaluate the quality of the proposed fully automated compartmentalization approach, we examined in which compartment the procedure places the reactions from the TCA cycle. In green algae, the TCA cycle, which oxidizes acetyl‐CoA to generate ATP, NADH, and FADH_2_, takes place in the mitochondria. The uncompartmentalized model includes nine reactions and 22 proteins associated with these reactions based on their GPR rules. By applying the compartmentalization pipeline, all reactions and associated proteins are correctly assigned to the mitochondrion compartment, thereby validating the proposed method for this pathway. Our initial findings indicated that careful curation of the protein localization is needed to ensure that the compartmentalization is in line with established knowledge. This is the case since errors in protein localization predictions propagate to the fully automated compartmentalization. To address this issue, as a part of the manual model curation, we introduced predefined reaction compartments for key metabolic pathways, including the Calvin–Benson cycle, photorespiration, and lipid metabolism, that must be respected in the optimization process. Future research will focus on preselection of protein localization predictions to minimize the need for human intervention.

Finally, the resulting *compartmentalized* constraint‐based model was augmented with enzyme constraints, yielding the eciCO1743 model. We also refined the model by adding atom mappings to facilitate future metabolic flux analyses (see ‘Constructing enzyme‐constrained model and atom mappings’ in the Materials and Methods section).

### Elimination of TICs

Metabolic models often contain reactions that can carry flux even if all uptake reactions are blocked, indicating the presence of TICs (see the Materials and Methods section and Supporting Information). To address this issue with the compartmentalized model, we identified a minimal set of reactions whose removal eliminates TICs while maintaining flux through the biomass reaction (see the Materials and Methods section and Supporting Information).

Due to differences in compartmentalization and solutions resulting from TIC elimination, different sets of reactions and genes are present under each growth condition. To address this issue, we integrated all models into a single consensus model, where each growth condition has its own biomass reaction and specific lower and upper bounds for fluxes. The final model, termed iCO1515, along with its enzyme‐constrained version, eciCO1515, is used in all subsequent analyses.

### Comparative structural analysis of the curated GEMs of *C. ohadii* and other green algae

Next, we assessed the structural properties of the curated iCO1515 model and compared them to those of phylogenetically close green algae GEMs, namely iCre1355 (Imam *et al*., 2015) of *C. reinhardtii*, iCZ843 (Zuñiga *et al*., 2016) of *C. vulgaris*, and iAJ526 (Juneja *et al*., 2016) of *C. variabilis*. The iCO1515 model includes 1515 genes, 3817 metabolites, and 2516 reactions (Table 1). At first glance, iCO1515 is larger than the compared models, primarily because it was reconstructed based on the entire databases of KEGG and MetaCyc, in contrast to the procedure that relies solely on the iRC1080 GEM of *C. reinhardtii* template model used in the reconstruction of the iCZ843 and iCre1355 models. As a result, iCO1515 can provide a more comprehensive insight into the pathways present in this algal species. Furthermore, transport reactions represent only 12.16% of the total reactions in iCO1515, which is lower than those in iCre1355 (18.8%), iCZ843 (16.5%), and iAJ526 (21.8%), in line with the principle used for the compartmentalization method employed in the iCO1515 reconstruction. We note that these transporters are not associated with GPR rules, due to the poor annotations of proteins with transport function; the procedure can readily be refined to retain such reactions if information is available.

**Table 1:** Comparison of structural properties between genome‐scale metabolic models (GEM) of green algae.

Property	iCO1515	iCre1355	iCZ843	iAJ526
Genes	1515	1460	843	531
Reactions	2516	2394	2294	1455
Reactions with GPR	1975	1924	1838	856
Transport	306	449	378	317
Exchange	10	11	15	14
Reversible	196	723	626	561
Irreversible	2320	1671	1668	894
Compartments	8	11	6	6
Metabolites	3517	1845	1770	1236
Unique metabolites	2539	1132	457	770

The table compares the key structural properties of our GEM iCO1515 of *Chlorella ohadii*, iCre1355 of *Chlamydomonas reinhardtii*, iCZ843 of *Chlorella vulgaris*, and iAJ526 of *Chlorella variabilis*. These include the number of genes; the number of reactions, grouped into enzymatic, transport, exchange, reversible, and irreversible; the total number of compartments; the number of metabolites; and the number of unique metabolites.

**Fig. 3:**
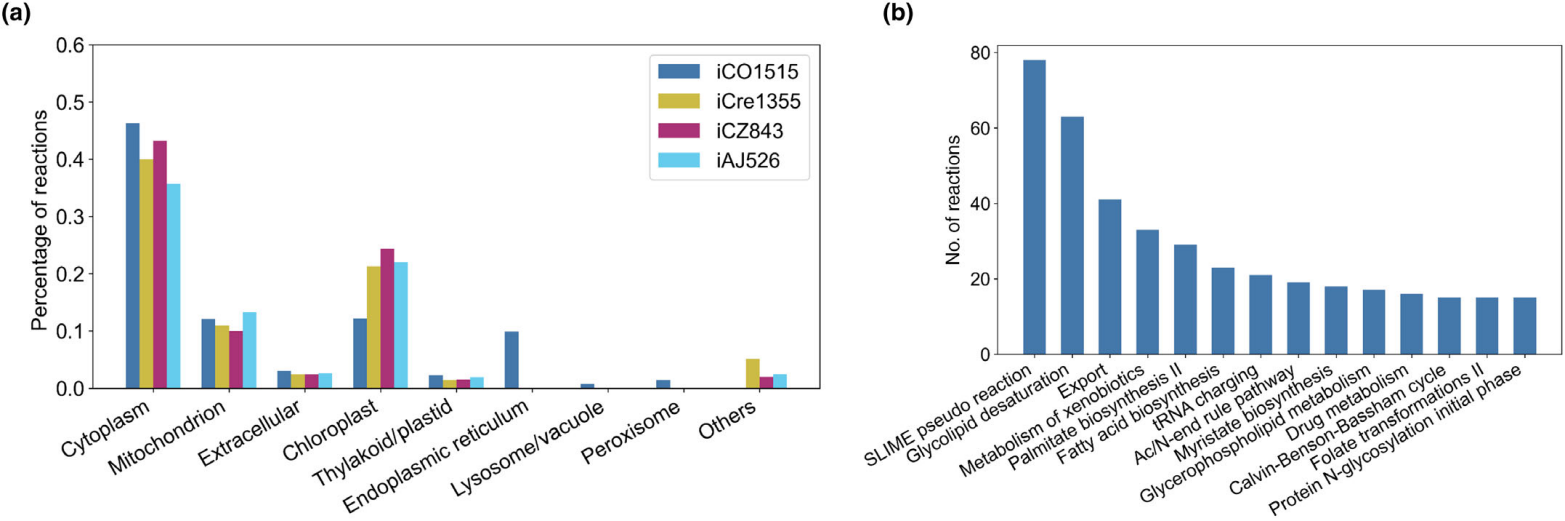
Comparison of structural properties of algal genome‐scale metabolic models (GEM) and the largest pathways of iCO1515. (a) The plot shows the percentage of the reactions in each compartment of the iCO1528 model of *Chlorella ohadii* compared with three other algal GEM. (b) The plot displays the top 12 largest subsystems of iCO1515 and their corresponding number of reactions.

The iCO1515 model includes eight compartments, in contrast to iCre1355, which contains 11 compartments, and iCZ843 and iAJ526, each with six compartments. Five compartments are shared between all of the compared models, namely cytoplasm, mitochondrion, chloroplast, thylakoid/plastid, and extracellular space, while three compartments are specific to our model, namely endoplasmic reticulum, lysosome/vacuole, and peroxisome (Fig. 3a). Despite these differences in the number of compartments, the percentages of reactions in the cytoplasm and mitochondrion are similar across all compared models (see Fig. 3a). The discrepancy in the percentage of reactions in the chloroplast is purely due to the technical separation of the reactions specific to the thylakoid in iCO1515.

The metabolic pathways included in iCO1515 with the largest number of reactions are part of lipid metabolism, such as the SLIME pseudoreactions (Sánchez *et al*., 2019), glycolipid desaturation, palmitate biosynthesis, myristate biosynthesis, glycerophospholipid metabolism, and fatty acid biosynthesis (Fig. 3b). We observed that the iCO1515 model also comprises a large number of export (41 reactions) and tRNA charging reactions (21 reactions), similar to the other compared models.

### Prediction of specific growth rates by the iCO1515 GEM is in line with experimental evidence

Next, we evaluated the functional capacity of the reconstructed iCO1515 GEM using data from growth experiments and compared its performance with GEMs of other green algae, as above. The biomass reaction of iCO1515 is composed of 48 biomass components, grouped into DNA, RNA, proteins, carbohydrates, chlorophylls, and lipids. The coefficients of the biomass precursors were determined using published experimental data (Treves *et al*., 2022) and genome‐based estimations, resulting in the generation of biomass reactions for four growth conditions, namely photoautotrophic low light, photoautotrophic high light, mixotrophic, and heterotrophic (see ‘Biomass reactions for different growth conditions’ in the Materials and methods section; Tables S1, S2). We ensured that the summation of the precursors' contribution weighted by their molecular weights amounts to 1 g g^−1^ DW, facilitating comparison of the predicted specific growth rates across the analyzed models.

**Fig. 4:**
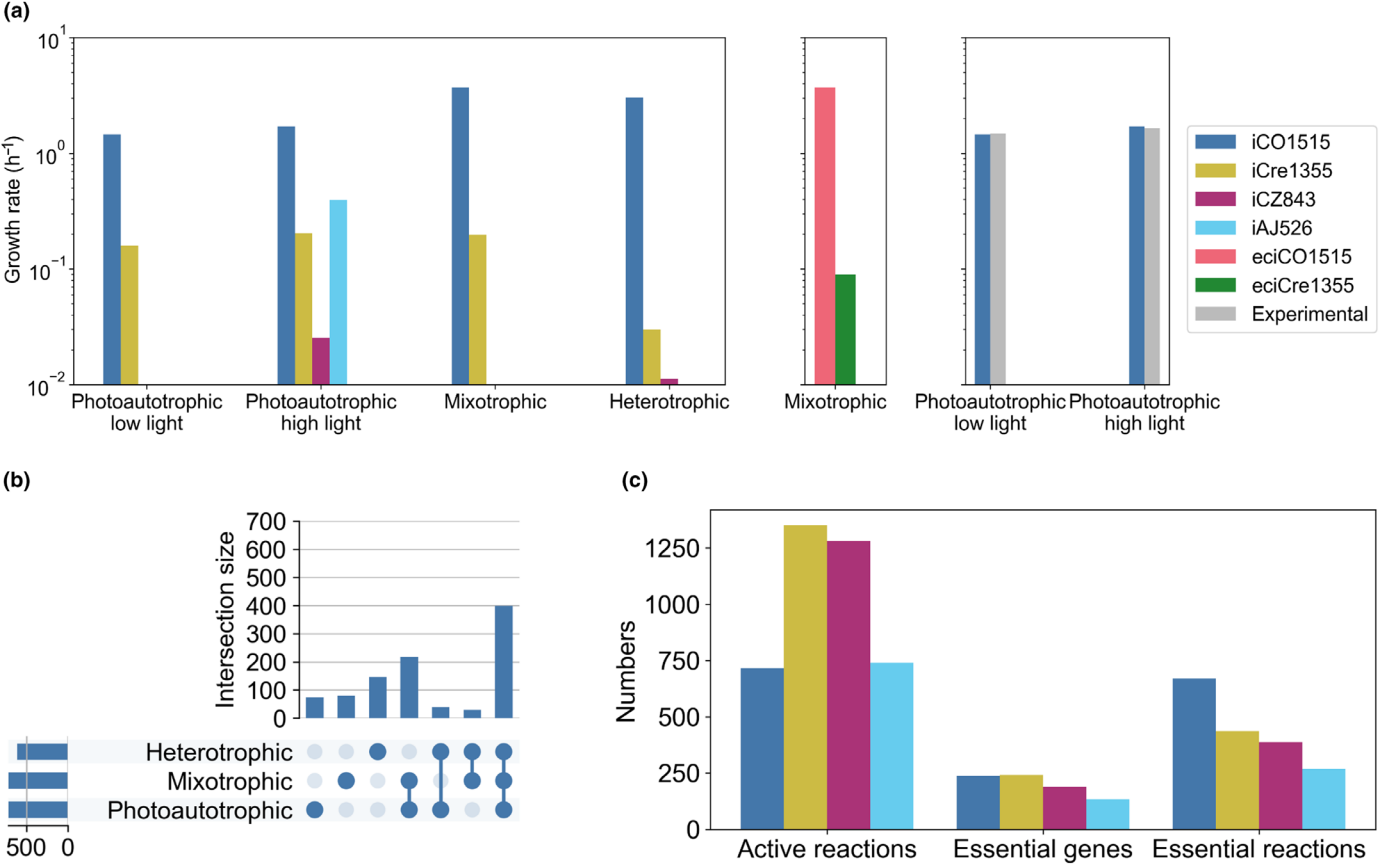
Comparison of specific growth rates simulated for different growth conditions. (a) (left) Specific growth rates predicted using FBA by iCO1515 of *Chlorella ohadii*, iCZ843 of *Chlorella vulgaris*, iCre1355 of *Chlamydomonas reinhardtii*, as well as iAJ526 of *Chlorella variabilis*, under photoautotrophic (low light: 100 μmol photons m^−2^ s^−1^; high light: 3000 μmol photons m^−2^s^−1^), mixotrophic (low light intensity, acetate, and CO_2_ as the carbon source), and heterotrophic conditions (acetate as the carbon source in the absence of light). For genome‐scale metabolic models (GEMs) that cannot predict specific growth rates for a given condition, either due to the absence of a condition‐specific biomass reaction or a blocked biomass reaction, the corresponding bars are omitted from the figure. (middle) Comparison of specific growth rates of enzyme‐constrained GEMs, namely eciCO1515 of *C. ohadii* and eciCre1355 of *C. reinhardtii* (Arend *et al*., 2023), in a mixotrophic condition. (right) Comparison of specific growth rates predicted by iCO1515 of *C. ohadii* with experimentally measured data from Treves *et al*. (2022) under photoautotrophic conditions at low light (100 μmol photons m^−2^ s^−1^) and high light (3000 μmol photons m^−2^ s^−1^) intensities. The *y*‐axis is logarithmically scaled for better display. (b) The UpSet diagram illustrating the distribution of active reactions across photoautotrophic, mixotrophic, and heterotrophic growth conditions. The sections containing two sets are specific to both and exclude the participation from the third set. (c) The panel illustrates the number of active reactions, essential genes, and essential reactions of iCO1515 of *C. ohadii* compared to iCre1355 of *C. reinhardtii*, iCZ843 of *C. vulgaris*, and iAJ526 of *C. variabilis*.

First, we compared the predicted specific growth rates using FBA (Orth *et al*., 2010) applied with the compared models for the four growth conditions (see Fig. 4a). For consistency, identical media were applied in all models by adjusting the lower bounds of exchange reactions to reflect the growth condition (see the Materials and Methods section). Our comparison was limited to growth rates simulated under photoautotrophic high‐light conditions, as some models (e.g. iCZ843 and iAJ526) predicted no growth under standard light conditions. We observed that the specific growth rate predicted by our model for the photoautotrophic high light growth condition (1.711 h^−1^) was the highest among all examined GEMs, showing 8‐, 67‐, and 4‐fold increase compared to those of iCre1355, iCZ843, and iAJ526, respectively. This prediction is consistent with experimental findings, showing that *C. ohadii* is the fastest growing green algae reported to date (Treves *et al*., 2022). Similarly, for the autotrophic low‐light, mixotrophic, and heterotrophic conditions, our model predicted growth rates of 1.459, 3.71, and 3.03 h^−1^, respectively, all of which exceeded the rates predicted by other algal GEMs.

We then compared the growth simulation of the enzyme‐constrained version of our model, named eciCO1515, with those of the *C. reinhardtii* ecGEM, eciCre1355, under the mixotrophic condition. For this, we applied identical chemostat conditions, with an acetate uptake rate of 2.72 mmol g^−1^ DW h^−1^, low light intensity (100 μmol photons m^−2^s^−1^), and a CO_2_ uptake rate of 2 mmol g^−1^ DW h^−1^. We found that eciCO1515 predicted a 41‐fold higher growth rate, consistent with predictions from conventional GEMs as well as the expected ranking of specific growth rates of these algae (see the second panel in Fig. 4a).

In addition, we assessed our model with the experimentally measured growth rates under two photoautotrophic conditions at low (100 μmol photons m^−2^s^−1^) and high (3000 μmol photons m^−2^s^−1^) light intensities (Treves *et al*., 2022) (see Fig. 4a). In these conditions, the predicted and experimental growth rates differ by less than 3%, demonstrating excellent agreement between our models and experimental measurements.

### Comparative analysis of functional properties between algal GEMs highlights the quality of iCO1515

To evaluate and compare the functional capabilities of the models, we performed flux variability analysis (FVA) (Mahadevan & Schilling, 2003) as well as analysis of synthetic knock‐outs, followed by enrichment analysis.

More specifically, we performed FVA to determine the feasible flux ranges for each reaction, enabling us to identify the active reactions in the model under each of the three growth conditions (Fig. 4b). To this end, each reversible reaction in the models was split into two irreversible reactions. Consequently, reactions with a positive flux range were identified as active reactions; in this analysis, we excluded the split reactions whose forward and backward directions had the same maximum flux value, to avoid false positive active reactions. We then identified reactions that are exclusively active in specific growth condition(s) (Fig. 4b). For instance, we identified 25 reactions that were active in both heterotrophic and mixotrophic conditions but were blocked under photoautotrophic conditions. These reactions, as expected, included acetate import (Table S4).

Notably, 142 reactions were exclusively active under heterotrophic conditions, compared to 71 and 76 reactions specific to photoautotrophic and mixotrophic conditions, respectively, which is in line with the additional pathways that enable growth in the absence of light and with the lower stacking of thylakoid membranes identified in *C. ohadii* cultures under carbon‐rich conditions (Treves *et al*., 2020). We found that over 39% of the 142 reactions specific to the heterotrophic growth condition are involved in lipid metabolism, energy production, and carbohydrate metabolism (Table S4). Specifically, these reactions included SLIME pseudo‐reactions (14 reactions), fatty acid biosynthesis (13 reactions), and glycolipid desaturation reactions (8 reactions), all in lipid metabolism, while the carbohydrate metabolism and energy production pathways comprised reactions in gluconeogenesis (9) and glycolysis (12).

Additionally, we performed gene and reaction essentiality analyses by simulating knockouts of individual genes and reactions to observe their impact on growth under photoautotrophic conditions in all compared models. We found that 237 of 1515 genes (15.64%) in the iCO1515 are essential for photoautotrophic growth (Fig. 4c). This proportion is similar to those of iCre1355 (16.57%), iCZ843 (22.3%), and iAJ526 (25.2%) under the same growth conditions. Moreover, 670 out of 2516 reactions (26.6%) in iCO1515 under photoautotrophic low light growth conditions are essential, which is greater than the corresponding proportions in other models (between 16.9% in iCZ843 and 18.4% in iAJ526). We also identified 168 essential genes and 376 essential reactions common across all growth conditions. These reactions are primarily involved in pathways related to the biosynthesis of essential fatty acids, amino acids, and nucleotides, as well as energy production and the regulation of metabolic intermediates, all of which are critical for cellular growth (Table S5).

Due to the differences in the reaction namespace between models, our comparative analysis was limited to comparing essential genes across the models. To this end, we conducted pairwise Blastp (Altschul *et al*., 1990) to compare the essential genes identified in our model with those in the iCre1355, iCZ843, and iAJ526 GEMs, under the photoautotrophic growth condition. Using a sequence alignment score with an E‐value below 0.01, we found 115, 78, and 164 homologous essential genes between our model and the iCre1355, iCZ843, and iAJ526 models, respectively. Our results identified 82 essential genes with no corresponding homologous genes in any of the compared models. Notably, 20% of reactions associated with these genes participate in lipid metabolism pathways, including glycolipid desaturation (24 reactions) and sulfolipid synthesis (5 reactions) (see Table S6). In particular, regulation of glycolipid desaturation, mediated by desaturase enzymes, plays a critical role in maintaining membrane homeostasis and functionality under stress conditions, such as high light and extreme temperatures typical for harsh habitats. We note that these results also demonstrate differences due to the incorporation of a larger number of lipid metabolism reactions in iCO1515 compared to the other algal models (Correa *et al*., 2020).

### Identification of target genes associated with high growth rates

Next, we sought to identify target genes that are associated with the higher growth rates of *C. ohadii*, based on the reference of the slower growing *C. reinhardtii*. To this end, we first divided the maximum growth rate of the GEM into 100 uniform increments. For each increment, we fixed the growth rate and computed the corresponding flux distribution using parsimonious flux balance analysis (pFBA) (Lewis *et al*., 2010), ensuring a very small optimal flux space. We then determined the correlation between the fluxes of each reaction and the specific growth rates, selecting reactions with a Pearson correlation coefficient greater than 0.8 and a *P*‐value below 0.01 (after multiple hypothesis correction). We then identified the unique set of genes associated with these reactions. This procedure was applied to both models, iCO1515 and iCre1355, under photoautotrophic conditions, resulting in the identification of 514 and 501 genes, respectively, whose corresponding reactions had flux correlated with growth rates. Similar to that mentioned previously, we also conducted a BLASTP analysis (Altschul *et al*., 1990) and identified 420 genes that are unique to our model and are potential candidates for modulating growth. We found that 7.7% of the reactions associated with these genes are involved in lipid metabolism pathways, including fatty acid biosynthesis (e.g. palmitate, myristate, and galactolipid, with 57 reactions) and glycolipid desaturation (55 reactions) (Table S7). Furthermore, 4.6% of these reactions are involved in folate transformations, which play a crucial role in nucleotide biosynthesis. In addition, 10.6% of the reactions contribute to carbohydrate metabolism, including glycolysis (43 reactions) and gluconeogenesis (23 reactions). These pathways are essential for synthesizing key carbohydrates, such as glucose, and breaking down carbohydrates to generate energy, which supports cellular growth.

Furthermore, we analyzed common enzymes in the GEMs of *C. ohadii* and *C. reinhardtii* that catalyze reactions with higher flux, leading to increased growth rates. To this end, we identified homologous enzymes in iCO1515 and iCre1355 by performing a BLASTP (Altschul *et al*., 1990) alignment with an E‐value threshold of 0.01. Consequently, we applied the optGpSampler (Megchelenbrink *et al*., 2014) flux sampling approach, generating 5000 flux distributions with a thinning constant of 1000 for each model. We then considered all possible reaction pairs catalyzed by the homologous genes and tested for significantly different mean sampled flux in iCO1515 compared to iCre1355, using a *t*‐test and Bonferroni‐adjusted *P*‐values. As a result, we identified 33 pairs of homologous genes, with corresponding reactions in iCO1515 exhibiting greater flux compared to those in iCre1355, and whose fluxes correlate with growth rate (Table S8). In line with the comparative flux analysis of these two algae (Treves *et al*., 2022), higher flux to catabolic energy metabolism pathways, including the TCA cycle and OPP, was predicted in *C. ohadii*. Notably, flux via 6‐Phosphogluconolactonase (Pgl) had a significant positive association with growth rate and was much higher in *C. ohadii*. As a key step in the oxidative phase of the OPP, this reaction has been shown to be crucial both for maintaining an efficient heterotrophic carbon flux, with Pgl overexpression resulting in *c*. 40% increased biomass under mixo‐ or heterotrophic conditions in *Phaeodactylum tricornutum* (Zhang *et al*., 2023), and for providing carbohydrates into the CBC to enhance CO_2_ fixation (Xu *et al*., 2022). Another major difference associated with the control of growth was a much higher predicted flux through Pyruvate phosphate dikinase (PPDK) in *C. ohadii*, in line with the higher flux into PEP in the latter (Treves *et al*., 2022). Other predicted differentially expressed reactions were associated with nucleotide metabolism and NAD^+^/NADP^+^ biosynthesis (Table S8). Further, several reactions were associated with regulatory pathways, including modifications of myo‐inositol‐containing compounds, which have been implicated in growth signaling in plants and algae (Gillaspy, 2011; Couso *et al*., 2016, 2021), or biosynthesis of the polyamine putrescine, previously shown to act as a positive growth signal in *C. ohadii*.

Finally, we identified the proteins in *C. ohadii* that catalyze reactions with higher flux values under high light conditions, potentially contributing to the resistance and increased growth of the green alga in its native environment. To this end, we took the intersection of the genes associated with at least one active reaction in both auto_100 and auto_3k growth condition models. Consequently, we generated 5000 flux distributions with a thinning constant of 1000 using optGpSampler (Megchelenbrink *et al*., 2014) for both models and identified genes whose corresponding reactions exhibit higher mean flux values in the auto_3k growth condition. As expected, the vast majority (52) of the 76 identified genes were predicted to be chloroplast localized, with a large number associated with PSII stability and function (Table S9), alongside several Calvin–Benson cycle enzymes, including PRK, TPI, PGK, and a CA1P phosphatase, which inactivates this Rubisco inhibitor (Andralojc *et al*., 2012). In line with the faster growth under high light, 22 of the identified reactions were associated with amino acid synthesis. This was accompanied by faster predicted flux through glycolytic and TCA cycle reactions, and notably, through the pyruvate–ferredoxin oxidoreductase (PFOR), which, similar to pyruvate dehydrogenase (PDH), decarboxylates pyruvate, but has been shown to be active under highly reducing conditions (Wang *et al*., 2022) when the latter is inactive. Significant increases were also predicted in several reactions associated with carotenoids, terpenes, and lipid biosynthesis and transport. These findings collectively demonstrate that the *in silico* comparative analysis based on fluxes between fast‐ and slow‐growing algae can raise new targets for improvement of growth, with evidence for some key enzymes already present.

## Discussion

Despite advancements in automating many steps of GEM reconstruction, including the usage of deep learning methods, achieving a fully automated pipeline for *de novo* GEM reconstruction for eukaryotic organisms remains a challenging task. Our study contributes to this ongoing progress in two ways: (1) by using the draft GEM, which is reconstructed solely based on databases instead of template models; and (2) by introducing a fully automated approach for compartmentalization. The proposed fully automated approach for compartmentalization assigns reactions to compartments using aggregated probabilities determined by subcellular location prediction tools. This is then followed by the MILP problem, which adds an optimum set of replicate and transport reactions to the GEM. This compartmentalization method, along with the automated procedure for removal of TICs, is the major innovation of our platform. We note that compartmentalization depends strongly on protein localization predictions; therefore, caution is warranted in directly using the resulting models without first inspecting the quality of the localization predictions – as illustrated in the case of the GEM for *C. ohadii*, which required careful inspection of the predictions. This limitation to full automation represents an active field of research and will be addressed in future studies.

Due to the limitations of current methodologies, particularly the absence of reliable automated methods for certain stages such as gap‐filling, we performed these steps using both automated methods and manual literature‐based curation. As a result, we provided well‐curated, functional GEMs and ecGEMs under three different growth conditions: photoautotrophic, mixotrophic, and heterotrophic. Comprehensive functional analysis and growth simulations were, in turn, performed with these models; the results were compared with those obtained from GEMs of other closely related green algae GEMs. Importantly, we demonstrated that the predictions from the developed model agreed with the experimental measurements of *C. ohadii*, supporting the experimental observations regarding the fastest growth reported to date for this alga compared to other algal species. Furthermore, our analysis reveals specific genes and pathways potentially conferring these high growth rates. We also integrated predicted *k*_cat_ and maximum enzyme pool into the GEM, constructed the ecGEM, and generated atom‐mapping for the reactions using the deep learning method, providing a comprehensive resource for future model‐driven systems biology studies of *C. ohadii*.

We envision that the semiautomated pipeline, along with future advances in the integration of machine/deep learning models, will provide the basis for generating pan‐genome‐scale metabolic models for algae (Arend *et al*., 2025). These advances are expected to facilitate more standardized comparative studies of the metabolic capabilities across diverse algal species.

## Competing interests

None declared.

## Author contributions

FS developed, implemented, and tested the approach, analyzed data, and curated models. SMC curated models and analyzed data. MA contributed to the development of the computational platform. HT gathered and analyzed data, and supervised students. NF interpreted findings from the modeling. ZR‐M contributed to the development of the computational platform. ZN designed the project, contributed to the development of the computational platform, acquired funding, and supervised students. FSB and ZN wrote the first draft of the manuscript. All co‐authors contributed to the final version of the manuscript.

## Disclaimer

The New Phytologist Foundation remains neutral with regard to jurisdictional claims in maps and in any institutional affiliations.

## Supporting information


**Methods S1** Gap‐filling.
**Methods S2** Compartmentalization.
**Methods S3** Elimination of thermodynamically infeasible cycles.

**Table S1** Biomass composition.
**Table S2** Biomass coefficients and gap fill details.
**Table S3** Details of subcellular localization tools.
**Table S4** Subsystem distribution of specific active reactions in the growth conditions.
**Table S5** Metabolic subsystems enriched with essential reactions under all growth conditions.
**Table S6** Metabolic subsystems enriched with essential genes specific to iCO1515.
**Table S7** Largest subsystems of the identified genes contributing to high growth rates specific to iCO1515.
**Table S8** Genes in iCO1515 with significantly higher fluxes in catalyzed reactions.
**Table S9** Genes of *Chlorella ohadii* with significantly higher fluxes under high light compared to low light growth conditions.
Please note: Wiley is not responsible for the content or functionality of any Supporting Information supplied by the authors. Any queries (other than missing material) should be directed to the *New Phytologist* Central Office.

## Data Availability

The FASTA files for *C. ohadii*'s genome are available at https://www.ncbi.nlm.nih.gov/datasets/genome/GCA_025026875.1/. The computational platform used for model generation and all procedures for downstream analysis are available at https://github.com/fayazsoleymani/c_ohadii_GEM.git to ensure reproducibility of the findings.
