# Supplementary material for: Constraint‐based metabolic modeling reveals metabolic properties underpinning the unprecedented growth of Chlorella ohadii
Source: New Phytol. 2025 Sep 5;248(3):1572–83. doi: 10.1111/nph.70528 (PMC12489276; doi:10.1111/nph.70528)
Supplement: Supplementary file 1 — Methods S1 Gap‐filling. Methods S2 Compartmentalization. Methods S3 Elimination of thermodynamically infeasible cycles. [file NPH-248-1572-s002.pdf]

***New Phytologist* Supporting Information**
Article title: Constraint-based metabolic modeling reveals metabolic properties underpinning the unprecedented growth of *Chlorella ohadii*
Authors: Fayaz Soleymani, Sandra Marcela Correa, Marius Arend, Niayesh Forghanisardaghi, Haim Treves, Zahra Razaghi-Moghadam, and Zoran Nikoloski
Article acceptance date: 08 August 2025

The following Supporting Information is available for this article:

Methods S1 Gap-filling
Methods S2 Compartmentalization
Methods S3 Elimination of Thermodynamically Infeasible Cycles

Methods S1 Gap-filling

First, for each metabolite in the biomass equation, an artificial demand reaction is added and its lower bound is set to a small positive value. We then utilized the entire KEGG model with the RAVEN function “gapfill” to add the smallest number of reactions to the model that ensure flux through the demand reaction. As a result, some of the biomass precursors could be produced in the network. The details are provided in Supplementary Table S2.

Regarding lipid metabolism, we expanded the network initially obtained from the draft reconstruction. To achieve this, we used the Plant Lipid Module created for *A. thaliana* as a reference (Cordoba et al., 2023; Li-Beisson et al., 2019), together with available information on the lipid composition of Chlamydomonas (Li-Beisson et al., 2015).This resulted in the addition of 47 new metabolites belonging to various lipid classes (*e.g.*, lysophosphatidic acid (LPA), phosphatidic acid (PA), diacylglycerol (DG), monogalactosyldiacylglycerol (MGDG), digalactosyldiacylglycerol (DGDG), phosphatidylglycerol (PG), sulfoquinovosyldiacylglycerol (SQDG), phosphatidylglycerophosphate (PGP), phosphatidylethanolamine (PE), phosphatidylinositol (PI)), along with 59 reactions that synthesize these lipids. Additionally, we included 78 pseudo-reactions following the SLIME approach described by (Sanchez et al., 2019),

which facilitates the incorporation of constraints on both lipid classes and acyl chain distributions, as well as the inclusion of lipid requirements in the biomass reaction. As a result, the synthesis of the lipid classes: LPA, PA, DG, MGDG, DGDG, PG, SQDG, PGP, PE, FA, PI, are integrated in the model. For the other biomass metabolites, manual curation was performed to add reactions to the model to resolve the gaps in pathways of the production of the corresponding metabolite.

For each added reaction, BLASTP (Altschul et al., 1990) was performed to identify orthologues in the genome of *C. ohadii* and to update the Gene-Protein-Reaction (GPR) rules.

Methods S2 Compartmentalization

First, location of each protein is predicted by employing five subcellular localization prediction tools, namely: BUSCA (Savojardo et al., 2018), DeepLoc 2.0 (Thumuluri et al., 2022), Localizer (Sperschneider et al., 2017), MuLocDeep (Jiang et al., 2021), and TargetP (Armenteros et al., 2019). Since each tool has its own distinct set of predicting locations, to unify the predictions, we considered the set of eight compartments to be included in the model. These compartments include the cytoplasm, mitochondrion, extracellular, chloroplast, thylakoid/plastid, endoplasmic reticulum, lysosome/vacuole, and peroxisome. Each tool yields a probability for the proteins in the intended compartments. If a tool does not predict probability for some of the aforementioned compartments, the probabilities of those compartments are obtained by subtracting the probabilities of all other compartments from one and then dividing the remaining probability by the respective count. For each of the tools, this procedure ensures the probabilities are normalized to have a summation equal to one (Supplementary Table S3). Subsequently, the probabilities for each protein for different compartments is aggregated from all the tools, and the protein is assigned to the compartment with the highest probability.

Having the localization prediction for each gene and GPR rule of each reaction, next reactions are allocated to the compartments using majority voting. For each reaction, the GPR rule is simplified to a set of proteins by removing the AND/OR operations. Each protein votes for a compartment and the votes are consequently normalized to percentages. Compartments with votes more than a specific threshold (we used 25%) are taken into account for each reaction. We refer to the copies of a reaction assigned in more than one compartment as “replicates”.

Furthermore, if a specific reaction is predicted to be present in more than one compartment, with corresponding replicates, the GPR rules of these reactions are modified. More specifically, the original GPR rule is simplified to its constituent genes that are then grouped to their corresponding compartment relying on the predictions. The GPR rule for each of the replicate reactions is generated based on the corresponding group of the genes. For the compartments which do not satisfy the threshold of the votes (here, 0.25) and has at least one gene in the group, their groups is merged to the most probable compartment so that no information is lost in the model.

Since in the compartmentalized model the identical metabolite may be present in discrete compartments, it is required to incorporate transport reactions between them. In this regard, we add all possible combinations with forward and backward direction between identical metabolites that are present in separate compartments.

Second, we intend to have a functional model with a minimum number of additional duplicate and transport reactions. To this end, we formulated a MILP to determine the smallest set of added reactions while respecting the model constraints:

$$
\begin{aligned}
\min_{\boldsymbol{v},\boldsymbol{d},\boldsymbol{t}} \quad & \lambda \sum_{i \in D} d_i + \sum_{j \in T} t_j \\
s.t. \quad & \boldsymbol{S}.\boldsymbol{v} = 0, \\
& d_i v_i^{lb} \leq v_i \leq d_i v_i^{ub}, \quad i \in D \\
& t_j v_j^{lb} \leq v_j \leq t_j v_j^{ub}, \quad j \in T \\
& v_k^{lb} \leq v_k \leq v_k^{ub}, \quad k \in R - (D \cup T) \\
& \sum_{l \in D_g; \forall D_g \subseteq D} d_l \geq \sigma, \\
& v \geq v_{bio}^{*}, \\
& d \in \{0,1\}^{|D|}, t \in \{0,1\}^{|T|}, v \in \mathbb{R}^{|R|}.
\end{aligned}
\tag{1}
$$

In this MILP formulation, D is the set of replicate reactions and $\boldsymbol{d}$ is a binary vector corresponding to each replicate reaction that gathers the statues of included / excluded reactions, T is the set of transport reactions and $\boldsymbol{t}$ is a binary vector corresponding to each transport reaction that collects their presence/absence in the solution. In addition, $\boldsymbol{S}$ denotes the

stoichiometric matrix, $\boldsymbol{v}$ represents a vector of the fluxes, each constrained in a range specified by the lower and upper bounds that are defined in the model. For each reaction with replicates, we define a subset D_g; therefore, we have a pairwise disjoint subsets whose union is the set D. For each of this subsets at least σ of their corresponding binary values from the vector $\boldsymbol{d}$ should be 1 (we used $\sigma = 1$ for our pipeline). Moreover, λ is a parameter that controls the trade-off between number of transport and duplicate reactions. By having $\lambda < 1$ we enforce that the solutions of the optimization problem favor the inclusion of more duplicate reactions compared to transport reactions and *vice versa* (we set $\lambda = 0.1$ in our pipeline). Finally, v^*_{bio} is the flux through the biomass reaction in which all of the duplicate and transport reactions are present in the model.

By solving the MILP problem we obtain a solution which consists of the binary vectors $\boldsymbol{d}$ and $\boldsymbol{t}$ alongside with a flux distribution $\boldsymbol{v}$. The variables which are equal to zero indicate that the corresponding replicate/transport reaction is not required for the functional model and could be removed. Consequently, these reactions are removed from the model. In addition, the genes in the GPR rules of the enzymatic replicate reactions which should be deleted, are moved to the most probable compartment of the reaction that would remain in the model.

Methods S3 Elimination of Thermodynamically Infeasible Cycles

First we identify the set of all reactions involved in TICs, by solving the following LP for $k \in R$, while all exchange reactions are blocked:

$$\begin{aligned} \max_{v} \quad & v_k \\ s.t. \quad & \boldsymbol{S}.\boldsymbol{v} = 0, \\ & v_i^{lb} \leq v_i \leq v_i^{ub}, \quad i \in R \\ & v_j^{lb} = v_i^{ub} = 0, \quad j \in E \\ & v \in \mathbb{R}, \end{aligned} \qquad (2)$$

in which E is the set of exchange reactions. Reactions that have a positive flux at the optimum in the LP are considered as the TICs, and we denote the set of all such reactions as C. Subsequently, we formulate a MILP to eliminate a minimal set of reactions from C, ensuring that these reactions cannot carry flux while simultaneously guaranteeing that the knocked-out

reactions do not eliminate biomass production. The former is achieved by considering the GEM with blocked uptake reactions, and the latter by allowing the GEM to uptake nutrients, while linking both models through the same knockout set:

$$
\begin{aligned}
\max_{\boldsymbol{v},\boldsymbol{v}',\boldsymbol{\delta}} \quad & \sum_{k\in C} v_k \\
s.t. \quad & \boldsymbol{S}.\boldsymbol{v} = 0 \\
& \delta_k v_k^{lb} \le v_k < \delta_k v_k^{ub}, && k \in C \\
& v_i^{lb} \le v_i \le v_i^{ub}, && i \in R - C \\
& v_j^{lb} = v_j^{ub} = 0, && j \in E \\
& \boldsymbol{S} \cdot \boldsymbol{v}' = 0, \\
& \delta_k {v'}_k^{lb} \le v'_k \le \delta_k {v'}_k^{ub}, && k \in C \\
& {v'}_i^{lb} \le v'_i \le {v'}_i^{ub}, && i \in R - C \\
& v'_{bio} > v'^{*}_{bio}, \\
& \sum_{k\in C} \delta_k = |C| - l, \\
& v \in \mathbb{R}^{|R|},\ v' \in \mathbb{R}^{|R|},\ \delta \in \{0,1\}^{|C|}.
\end{aligned} \tag{3}
$$

In this optimization problem, l denotes the number of knock-outs, and v'^{*}_{bio} is the optimum biomass from FBA. We solve the MILP iteratively for $l = \{0, 1, \cdots n_C\}$ and check the objective value. The process terminates in the iteration where the maximum sum of fluxes in the TICs equals zero, yielding the set of reactions that should be removed from the model.

References

S. C. C´ordoba, 606 H. Tong, A. Burgos, F. Zhu, S. Alseekh, A. R. Fernie, and Z. Nikoloski. Identification of gene function based on models capturing natural variability of arabidopsis thaliana lipid metabolism. *Nature communications*, 14(1):4897, 2023.

Y. Li-Beisson, J. J. Thelen, E. Fedosejevs, and J. L. Harwood. The lipid biochemistry of eukaryotic algae. *Progress in lipid research*, 74:31–68, 2019.

Y. Li-Beisson, F. Beisson, and W. Riekhof. Metabolism of acyl-lipids in chlamydomonas reinhardtii. *The Plant Journal*, 82(3):504–522, 2015.

B. J. Sanchez, F. Li, E. J. Kerkhoven, and J. Nielsen. Slimer: probing flexibility of lipid metabolism in yeast with an improved constraint-based modeling framework. *BMC systems biology*, 13:1–9, 2019.

S. F. Altschul, W. Gish, W. Miller, E. W. Myers, and D. J. Lipman. Basic local alignment search tool. *Journal of molecular biology*, 215(3):403–410, 1990.

C. Savojardo, P. L. Martelli, P. Fariselli, G. Profiti, and R. Casadio. Busca: an integrative web server to predict subcellular localization of proteins. *Nucleic acids research*, 46(W1):W459–W466, 2018.

V. Thumuluri, J. J. Almagro Armenteros, A. R. Johansen, H. Nielsen, and O. Winther. Deeploc 2.0: multi-label subcellular localization prediction using protein language models. *Nucleic acids research*, 50(W1):W228–W234, 2022.

J. Sperschneider, A.-M. Catanzariti, K. DeBoer, B. Petre, D. M. Gardiner, K. B. Singh, P. N. Dodds, and J. M. Taylor. Localizer: subcellular localization prediction of both plant and effector proteins in the plant cell. *Scientific reports*, 7(1):44598, 2017.

Y. Jiang, D. Wang, Y. Yao, H. Eubel, P. Kunzler, I. M. Moller, and D. Xu. Mulocdeep: a deep-learning framework for protein subcellular and suborganellar localization prediction with residue-level interpretation. *Computational and structural biotechnology journal*, 19:4825–4839, 2021.

J. J. A. Armenteros, M. Salvatore, O. Emanuelsson, O. Winther, G. Von Heijne, A. Elofsson, and H. Nielsen. Detecting sequence signals in targeting peptides using deep learning. *Life science alliance*, 2(5), 2019.
